# Cases of Uræmia as Sequela of Scarlet Fever

**Published:** 1862-05

**Authors:** W. Brooks

**Affiliations:** Chicago


### CASES OF URÆMIA AS SEQUELA OF SCARLET FEVER.

By W. BROOKS, M.D., of Chicago.

Was called at 10 o'clock, A. M., May 10th, 1851, in consultation with the late Dr. — to H. S. of N—, aged two years. Found her skin pale, dry and harsh, general anasarca, pulse sixty small, does not swallow, comatose, pupil of eyes dilated to greatest extent, a slight convulsion occasionally, decided ammoniacal odor in her breath, has passed no urine for forty hours, evidently in articulo mortis; died ten minutes past eleven. The following are the details of the case as narrated by Dr. L.: About three weeks previously she was attacked very lightly with simple scarlatina, desquamation commenced on the sixth day of the eruption and progressed apparently favorable until eleven days after the first appearance of desquamation and the twenty-first from the attack; when the mother discovered a general fullness that was unnatural and her urine small in quantity, smoky and of a port wine color, the child sleepy and snored very loudly, could be roused, but at once relapsed. She was confident the child had taken cold the day previous. At this time, Dr. L. was called and prescribed as follows: ℞—Bitartrate Potassa i ʒ; Aqua ss O. Solve to be drank in twenty hours. ℞—Buchu Fol. i ℥; Aqua Bullient, i ʒ. Infuse; give one dram every three hours. This treatment was continued, the urine diminishing until it was suspended about four o'clock A. M. the 9th, being about forty-one hours previous to decease, after which she was roused with great difficulty. Fifteen hours before death, convulsions supervened.

By request, sectio cadaver was had twenty-four hours after death. General appearance, sanguine, anasarcous; brain, the membranes paler than natural, slight effusion between the arachnoid and dura mater, and in the ventricles. As the calvarium was being removed, a decided urinous odor was emitted. One dram of the fluid was preserved, which, being put in a flask and heat applied, gave a strong ammoniacal odor. The lungs were somewhat engorged with blood; there were two ounces of nearly transparent fluid in the chest. The same urinous odor was observed when we opened the cavity of the chest as when the calvarium was removed, and a similar ammoniacal one emitted on application of heat to the fluid. On opening the abdomen, the urinous odor was still more marked· Here we found about six ounces of a pale-straw colored fluid, to which heat being applied, the same result was obtained as from that of chest, etc. There was nothing remarkable about the heart. Stomach, intestines, liver and spleen not unnatural, except they were abnormally pale; bladder entirely empty, except two or three casts of tubuli uriniferi. Kidneys very much congested and filled with epithelium. In the pelvis of the left kidney were a few drops of urine, which, on analysis, gave the same results as one dram did which was passed forty-one hours previous to death (which was the last and all that was then passed, and had been carefully preserved by the mother). This proved albuminous by application of heat and nitric acid. Under the microscope could be seen good casts of the tubuli uriniferi, epithelial cells and blood corpuscles.

*Case 2d.*—Was sister of preceding, four years old. Was summoned to meet Dr. P. in consultation, at six o'clock A. M. the 13th. She had had a similar attack of scarlatina at the same time as her sister. Found her pale, anasarcous, comatose, breathing stertorous, pupils dilated and immobile, breath ammoniacal, pulse fifty-eight, feeble, skin dry and very harsh; has occasional spasms; has passed no urine since four o'clock P. M. the day previous, and then only two drams; can only be roused for a single instant. Introduced a catheter; found the bladder empty. She was evidently sinking, and died at eight o'clock the same morning. She was exposed to cold at the same time as her sister; but no symptoms that caused her parents to send for their physician, Dr. P., until twelve o'clock M. of the 12th. He gave ℞—Calomel iii grs.; Jalap iii grs., which was to be repeated in six hours, unless it proved actively cathartic; also, ℞—Buchu Fol. i ℥; Aquæ Bullient i℈; infuse, give two drams every three hours. The urine was suspended about four o'clock P. M. of the same day. Sectio cadaver twenty-six hours after death. The post-mortem appearances were nearly identical with the sister. The only difference we were able to detect was a little urine in the pelvis of both kidnies. There was the same urinous smell on opening the cavities, and the same ammoniacal odor evolved on subjecting the fluid of the cavities to the action of heat, and the microscope revealed a similar condition of urine.

Was called to Ida B——, at eight o'clock A. M., March 11, 1860, aged four and a half years, and to Jenny B——, her sister, at five o'clock P. M. of same day, aged three years and two months. It was the first day of the eruption of very mild cases of Scarlatina. The bowels being slow gave Ida, ℞—Calomel iii grs., Ipecac Pulv. 1 gr. In six hours, it not having moved the bowels, gave ℞—Fluid Ext Senna ss ℥; the throat symptoms were scarcely observable, a free use of diluent drink being all that that was required. To Jenny gave, ℞—Calom! ii grs., Pulvis Ipecac i gr. It acted freely as a cathartic in five hours. To her also gave freely of diluents. Desquamation commenced on the fifth day of the eruption and progressed slowly. It seemed more like a throwing off of branny scales, or more properly epithelial scales, rather than a cuticular desquamation. They each had a warm bath once per diem, with directions that they be kept in their room and well protected.

On the morning of March 21st, saw them and both were apparently doing well. A six o'clock P. M. was called hurriedly to them, and on arrival found that, contrary to positive directions, the day being very fine the nurse had taken both children into the door-yard for about two hours, and alternately drawn one in a hand carriage while the other stood on the ground. Ida, the eldest, presented the following aspect: generally anasarcous; skin elastic, with bright red cheeks; comatose. Soon after my arrival, passed one ounce of urine, smoky in looks, of peculiar red color, which under the microscope exhibited blood corpuscles, casts of tubuli uriniferi and epithelial cells. On applying heat to it, it yielded Albumen; also the same on the addition of Nitric Acid. Bowels had moved naturally at 8 A. M., pulse sixty-four, pupils dilated, and would not contract by placing a lighted lamp near them; skin dry and harsh. ℞—Calomel x grs., Pulv. Ipecac iiss grs. M.—ft Chart No. V; one to be given every two hours. ℞—Citrate of Potassa (officinal solution) i ℥, Aqua Pura ss O. M—Give one tablespoonful every two hours. A warm bath.

5 A. M. Bowels have not acted; has passed one dram of urine like that of yesterday on analysis except darker in color. Still comatose, pupils dilated to maximum, immobile, can be roused, but instantly relapses, thirsty, skin intensely hot and dry, pulse fifty-four, anasarca increasing, peculiar bright color of one cheek, an unusual ammoniacal odor in breath. Mustard semicupium sinapism over the kidneys, heated before applying; repeat Calomel and Ipecac; continue Citrate Potassa. ℞—Triticum Repens ss ℥, Aquæ Bullient ss O.; infuse one hour; one ounce to be given every three hours.

6 P. M. Met Dr. L—— in consultation.

4th. Last hour has been restless, bowels have not been moved, no urine passed, skin very harsh, pupils still dilated and immobile; can be roused, but with difficulty; ammoniacal fœtor in breath increased. Continue treatment. Eleven P. M. At half-past ten bowels acted freely; sinapism had also acted; seemed to be fretted by it; pupils still dilated; can detect no urine in bladder. At half-past eleven, complained of something hurting her in the region of the kidneys. Three minutes later, of pain in or near the bladder, and very soon a desire to urinate, and soon passed two ounces of highly albuminous urine, charged largely with epithelial cells, casts of tubuli uriniferi and blood corpuscles. Bowels moved freely twenty minutes of twelve. At twelve o'clock passed three ounces of urine, with less abnormal elements in it, and immediately a profuse perspiration broke forth, in truth she was literally bathed in it, which emitted the odor of warm urine. Suspend Calomel and Ipecac; continue Citrate Potassa and Triticum Repens. At one o'clock A. M. she passed three and half ounces of urine much lighter colored. At two A. M. passed yet more and waked up feebly. At this time the pupil contracted partially. Six o'clock A. M.—Has passed urine copiusly about once an hour near four ounces at a time on the average; perspiration abating; pupils contract quite freely; pulse eighty; is bright and lively; bowels have moved copiously; anasarca much abated. Twelve M—Urine very copious, light pink color, very little albumen; calls for food; give black tea and toast bread; continue Triticum Repens. Six P. M.—Evidently improving; pulse eighty-five; bowels moved freely at five P. M.

24th.—Ten A. M. Improving. Continue diet and treatment.

25th.—From this time for six days she continued to improve, when there was still some blood corpuscles in the urine. ℞—Tinct. Mur Ferri 1 ℥; M. Liquid Hydrochloric Acid xxx minims. Three gtts. in water every four hours. Of the infusion of Triticum Repens, one dram every three hours.

26th.—Ten A. M. Improving; continue treatment.

27th.—Ten A. M. Much improved. The Sesquichloride of Iron was continued three days longer, when neither albumen, casts of tubules or blood corpuscles were discernible; treatment was suspended; desquamation was soon completed, and now, April 5th, 1862, is in perfect health.

March 21st.—6 P. M. Found Jenny in convulsions, pupils dilated, immobile, skin dry and harsh, pulse sixty-one, heavy, laboring, breathing stertorous, insensible; has, to the best of nurse's belief, passed no urine since ten A. M.; repeated efforts at vomiting; odor of orange peel in her mouth; by irritating the fauces with a feather she vomited perhaps one eighth part of the peel of a small orange, with some nearly colorless fluid. Convulsions soon abated, anasarcous, the flesh having a peculiar elastic feel. ℞—Calomel viii grs., Ipecac Pulv. ii grs. M. Chart, No. V. Give one every two hours. ℞—Citrate Patassa (of. sol.) iss℈, Aqua Pura ss O. M—Give one half ounce every two hours hot pediluvium with mustard; hot sinapism over the kidneys three by six inches.

22d, 5 A. M.—Bowels have acted moderately; no urine passed; can detect none in the bladder with catheter. No convulsions since 11 P. M. yesterday; comatose, pupils still dilated and immobile, can be roused but very little. Repeat calomel and ipecac; continue Citrate of Potassa. ℞—Triticum Repens, ss℥; Aquæ Bullient, ss℈. Infuse one hour; strain. Give one ounce every three hours. Apply blister three by six inches over the region of the kidneys.

At 6 P. M., met Dr. L. in consultation. Blister has acted well; when vesication took place, she aroused some, which was at 2 P. M.; at half-past two, she cried out as with pain, and applied both hands over the renal organs for a moment, and then transferred them to the hypogastrium, pressing and crying for a short time. At 3 P. M., she passed two ounces of urine of dark port wine color; analysis by heat and nitric acid showed albumen largely. The microscope, epithelial cells, casts of tubuli uriniferi and blood corpuscles. 7 P. M., is perspiring freely, with strong urinous odor. At half-past 7, passed five ounces of urine of much lighter color, which yielded some albumen, many casts of tubuli and some blood corpuscles; is bright and cheerful, disposed to be wakeful. Bowels have moved copiously four times during the last three hours. Desires a little food. Give some coffee made of unleavened bread roasted. Continue triticum repens.

23d, 10 A. M.—Greatly improved; urine has very little albumen, a few casts and blood corpuscles. 24th, urine appears well, except some blood corpuscles. On the 27th, some blood corpuscles remaining, ordered ℞—Tinct. Mur. Ferri, i℈; Liquid Hydrochloric Acid, v gtts. M.—Give three gtts. once in six hours.

29th.—All abnormal appearances had gone from urine; with a mild diet, she was soon convalescent, and is now, April 7th, 1862, in good health.

Feb. 28th, 1862, 8 o'clock P. M.—Was called to W. H—s, of Peoria, aet. seventeen years. He reports that a month ago he had a light attack of scarlet fever at Peoria; he came from thence to this place three days since. Very sleepy, rouses without much difficulty; the pupil of the eyes dilated to more than one half the extent of the cornea. The finger applied close to the eye will not cause the pupil to contract, and the same with a lamp. Pulse fifty, full, heavy, and labored. Skin hot, dry, harsh and sallow; general anasarca; flesh very elastic; breathing, when asleep, stertorous. Has passed about one dram of urine since morning, of a brownish red color, smoky appearance. Yields albumen, casts of tubuli uriniferi and blood corpuscles. Desquamation appears to be about one-half completed. ℞—Calomel, xx grs.; Ipecac Pulv. v grs. M. Chart No. IV—Give one every two hours. ℞—Citrate Potassa (Off. Sol.) i ℥; Aquæ Pura, i O. Give one and a-half ounces every two hours. Pediluvium of very hot mustard and water.

March 1st, 8 o'clock A. M.—Has slept and breathed very heavily all night; skin dry and harsh, pulse sixty-five; thirsty, tongue covered with a thick yellow coat, pupils still dilated immobile, bowels have not yet acted, urine two ounces since last evening, very much like that of yesterday. Repeat Calomel and Ipecac if it does not act briskly within two hours of taking last powder given. ℞ Fluid Ext. Senna ss. ℥; also, ℞ Bitartrate Potasse ss. ℥; Aquæ Bullient i O. Give as a drink ad libitum. Seven o'clock P. M. Bowels have acted copiously, urine increased to about ten ounces. Abnormal appearances much the same. Anasarca not changed. Sleeps most of the time and heavy pupils unchanged, Desquamation recommencing, continue Citrate and Bitartrate Potassa Diet gruel, repeat pediluvium.

March 2d. Eight o'clock A. M. Still sleepy, skin dry, pupils contract some, dejections from bowels very copious, urine largely increased, remains of a dirty port wine color. Continue treatment. Eight P. M. Improved, less drowsy.

3d. Nine A. M. Urine very free, bowels have acted twice this morning freely. Anasarca disappearing, abnormal products of urine less. Eight and a half P. M. Very comfortable, more wakeful, pulse sixty-eight. Continue treatment, light diet.

4th. Appears to be improving, desquamation going on, continue treatment. Nine P. M. Sleeps too heavily, otherwise seems to be doing well, has been more wakeful during the day.

5th. 9 A. M. Much as yesterday, some unnatural dilation and immobility of pupil. Diet, stale toast bread and black tea. Continue treatment. 7 P. M. Sleeps too much.

6th. Anasarca much abated. Urine is yet albuminous. Some tube casts and some blood corpuscles, but is quite free. Skin is still dry. Sleeps less heavy, bowels have moved freely. Continue treatment. 9 P. M. Very bright.

7th. 8 A. M. At two o'clock this morning, he awoke with most intense head-ache, which lasted at least five hours and then gradually abated. Appears bright, pupils contract pretty well. Skin dry, pulse eighty-two; bowels moved freely at 7½ this morning. Urine free, has abnormal products in it to some extent. 9½ P. M. Is now asleep, breathes heavily, very heavily; his brother thinks not more so than is natural to him. Fifteen minutes of ten, is awake, says he has no headache, feels pretty well. Says no one can imagine the intensity of his suffering with his head for about two hours this morning. He soon fell asleep. The headache returned about twelve o'clock.

8th. 1½ A. M. Summoned to him in haste. Found him in a sitting posture in bed, rigid, with a generally convulsed condition. Eyes closed, no deglutition. Sinapism eight by twelve inches over the kidnies. Sinapisms to wrists and ancles. He gradually relaxed so far as to be placed in a recumbent posture.

2½ A. M.—Paralysis of left half of body. Sinapisms have not acted; has not swallowed. 3¼ o'clock. Some motion of left hand. 4 A. M. Right side paralyzed. Very little motion of left. Pulse for the last hour forty-two. 4½ o'clock. Sinapism acting smartly. Convulsions abating; he made a little groan for the first time. 5 o'clock. Emplastrum Cantharis five by twelve inches, applied on the place from whence the sinapism was removed. Some urine has just passed involuntarily.

7 A. M.—Convulsions have ceased; at six, manifested a desire to urinate, was assisted and passed about five ounces of smoky urine, much like that passed the 6th. Vesication is taking place. Can swallow without much difficulty. Paralysis of right side abating.

℞—Calomel xx grs. Ipecac pulv. v grs. M. f. t. Chart No. iv. One every two hours. ℞—Triticum Repens. i ℥ Aquæ Bullient, i O. Infuse one hour, give two ounces every three hours.

9 A. M.—Profuse perspiration with strong urinous odor and ammoniacal odor in breath. Vesication complete, blister to be dressed with leaves of cabbage dipped in hot water. Describes the approach of the convulsions as a drawing of the head from the pillow upward, as he was waking from sleep, till he came erect in bed when he lost consciousness, and it was in this state I found him.

6 P. M.—Bowels have not acted; sweats freely; urine free; pupil contract well; desquamation is proceeding rapidly. Repeat Calomel and Ipecac. Continue Triticum Repens. Very light diet.

10th.—Decidedly improving. Continue treatment.

11th.—9 A. M. Decidedly convalescing. The abnormal elements disappearing, blood corpuscles more in quantity. ℞—Mur Tinct. Ferri. i ℥; Liquid Hydrochloric Acid ss ℨ. M—Give ten g tts. once in six hours. Continue Triticum Repens. Under this treatment he continued to improve till the 22d, when he seemed convalescent. On the 25th he exposed himself to fatigue, and on the the 26th there was some Hæmaturia. ℞—Triticum Repens. i ℥. Aquæ Bullient i O. Infuse one hour, take two ounces every three hours. Take of the preceding formula of Sesquichloride of Iron ten g tts. every six hours, and light farinaceous diet; this was pursued a few days and very slight hœmaturia remaining. ℞—Acidum Gallicum, xxi. grs. F. t. Chart No. 7. One every four hours. Under this all abnormal constituents disappeared from the urine. And now, April 14th, 1862, all diseased action seems to have passed away.

Elaborate microscopic detail, or elegantly wrought theories will hardly be anticipated of one engaged in the active duties of professional life. Believing the above related cases to be those of Uræmic intoxication or poison, as the result of exposure to cold during the period of desquamation in cases of simple scarlatina, we may venture the following deductions. We have two or three conditions present, which almost entirely lead to the train of symptoms observed in each case: First, the skin was dry and harsh, preventing such cutaneous elimination of the scarlatinal poison, as would have been the case if desquamation had been free and the skin had returned early to a normal state. Second, at the Sectio Cadaver the kidnies were in a very analogous condition to that of the skin. As there has been a desquamative state of the one, so there has been of the other. The kidney was filled with epithelium, the organ consequently enlarged, and in a state of hyperæmia as far as this filling with epithelium will permit. Thus, there is not only a large quantity of blood in the organ, but it is irregularly distributed and there is an undue quantity of epithelium in the tubuli uriniferi. This accumulation of epithelium over distends the tubes, thereby compressing the small vessels, which ramify upon the walls. Thus the blood is thrown back on the Malpighian bodies and the vascular system is irregularly supplied. When, hence, hæmaturia, with little or no secretion of urine, the blood being often exosmosed from the small vessels that are unduly filled. When congestion of the Malpighian bodies exists to a great degree it leads to the rupture of the Malpighian vessels and the escape of all the constituents of the blood. Hence, we find in the urine albumen, blood corpuscles, casts of tubuli, etc., which give to the urine its peculiar color and smoky appearance. It will be observed that the cases had progressed well up to the given point, that they had been very light, or in other words there had been very little scarlatinal irritation of the skin and the desquamation imperfect, when on exposure to cold the cutaneous functions were suspended, causing an increased determination of blood to the already disordered kidnies. This disorder arising from imperfect desquamation and filling of the tubes with epithelium, The urine, therefore, is not secreted at all or very little, it remains consequently in the blood and uræmic intoxication is the result. Without proceeding in detail with an argument we will conclude by saying, that the character of the urine and its secretion, the state of the kidnies as observed, the condition of the pupil and the character of the coma make it patent to the writer that that they were each and all cases of uræmia, and of the grave sequlæ of scarlatina.

